# Disruption of PHF21A causes syndromic intellectual disability with craniofacial anomalies, epilepsy, hypotonia, and neurobehavioral problems including autism

**DOI:** 10.1186/s13229-019-0286-0

**Published:** 2019-10-22

**Authors:** Hyung-Goo Kim, Jill A. Rosenfeld, Daryl A. Scott, Gerard Bénédicte, Jonathan D. Labonne, Jason Brown, Marianne McGuire, Sonal Mahida, Sakkubai Naidu, Jacqueline Gutierrez, Gaetan Lesca, Vincent des Portes, Ange-Line Bruel, Arthur Sorlin, Fan Xia, Yline Capri, Eric Muller, Dianalee McKnight, Erin Torti, Franz Rüschendorf, Oliver Hummel, Zeyaul Islam, Prasanna R. Kolatkar, Lawrence C. Layman, Duchwan Ryu, Il-Keun Kong, Suneeta Madan-Khetarpal, Cheol-Hee Kim

**Affiliations:** 1 Neurological Disorders Research Center, Qatar Biomedical Research Institute, Hamad Bin Khalifa University, Doha, Qatar; 2 Department of Molecular and Human Genetics, Baylor College of Medicine, Houston, TX USA; 3 Department of Molecular Physiology and Biophysics, Baylor College of Medicine, Houston, TX USA; 4 Laboratoires de Diagnostic Génétique, Unité de génétique moléculaire, Nouvel Hôpital Civil, Strasbourg Cedex, France; 5 Section of Reproductive Endocrinology, Infertility & Genetics, Department of Obstetrics & Gynecology, Augusta University, Augusta, GA USA; 6 Baylor Genetic Laboratories, Houston, TX USA; 7 Kennedy Krieger Institute, Baltimore, MD USA; 8 Department of Medical Genetics, Lyon University Hospital, Lyon, France; 9 Department of Pediatric Neurology, Lyon University Hospital, Lyon, France; 10 Équipe Génétique des Anomalies du Développement (GAD), INSERM, Dijon, France; 11 Centre de Génétique, CHU Dijon Bourgogne, Dijon, France; 12 Service de Génétique Clinique, CHU Robert Debré, Paris, France; 13 Clinical Genetics, Stanford Children’s Health at CPMC, San Francisco, CA USA; 14 GeneDx, Gaithersburg, MD USA; 15 Max Delbrück Center (MDC) for Molecular Medicine, Berlin, Germany; 16 Diabetes Center, Qatar Biomedical Research Institute (QBRI), Hamad Bin Khalifa University, Doha, Qatar; 17 Department of Neuroscience and Regenerative Medicine, Augusta University, Augusta, GA USA; 18 Department of Statistics and Actuarial Science, Northern Illinois University, DeKalb, IL USA; 19 Department of Animal Science, Division of Applied Life Science (BK21plus), Gyeongsang National University, Jinju, Korea; 20 Pediatric Medical Genetics, Children’s Hospital of Pittsburgh, Pittsburgh, PA USA; 21 Department of Biology, Chungnam National University, Daejeon, Korea

**Keywords:** *PHF21A*, *BHC80*, Intellectual disability (ID), Autism spectrum disorder (ASD), Neurodevelopmental disorders, Potocki-Shaffer syndrome (PSS), AT Hook domain, Intrinsically disordered region (IDR), *KDM1A*

## Abstract

**Background:**

*PHF21A* has been associated with intellectual disability and craniofacial anomalies based on its deletion in the Potocki-Shaffer syndrome region at 11p11.2 and its disruption in three patients with balanced translocations. In addition, three patients with de novo truncating mutations in *PHF21A* were reported recently. Here, we analyze genomic data from seven unrelated individuals with mutations in *PHF21A* and provide detailed clinical descriptions, further expanding the phenotype associated with PHF21A haploinsufficiency.

**Methods:**

Diagnostic trio whole exome sequencing, Sanger sequencing, use of GeneMatcher, targeted gene panel sequencing, and MiSeq sequencing techniques were used to identify and confirm variants. RT-qPCR was used to measure the normal expression pattern of *PHF21A* in multiple human tissues including 13 different brain tissues. Protein-DNA modeling was performed to substantiate the pathogenicity of the missense mutation.

**Results:**

We have identified seven heterozygous coding mutations, among which six are de novo (not maternal in one). Mutations include four frameshifts, one nonsense mutation in two patients, and one heterozygous missense mutation in the AT Hook domain, predicted to be deleterious and likely to cause loss of PHF21A function. We also found a new C-terminal domain composed of an intrinsically disordered region. This domain is truncated in six patients and thus likely to play an important role in the function of PHF21A, suggesting that haploinsufficiency is the likely underlying mechanism in the phenotype of seven patients. Our results extend the phenotypic spectrum of *PHF21A* mutations by adding autism spectrum disorder, epilepsy, hypotonia, and neurobehavioral problems. Furthermore, *PHF21A* is highly expressed in the human fetal brain, which is consistent with the neurodevelopmental phenotype.

**Conclusion:**

Deleterious nonsense, frameshift, and missense mutations disrupting the AT Hook domain and/or an intrinsically disordered region in PHF21A were found to be associated with autism spectrum disorder, epilepsy, hypotonia, neurobehavioral problems, tapering fingers, clinodactyly, and syndactyly, in addition to intellectual disability and craniofacial anomalies. This suggests that *PHF21A* is involved in autism spectrum disorder and intellectual disability, and its haploinsufficiency causes a diverse neurological phenotype.

**Electronic supplementary material:**

The online version of this article (10.1186/s13229-019-0286-0) contains supplementary material, which is available to authorized users.

## Background

The Potocki-Shaffer syndrome (PSS [MIM 601224]) is characterized by intellectual disability (ID), craniofacial anomalies, multiple exostoses, and biparietal foramina. It is a contiguous gene deletion disorder caused by haploinsufficiency of multiple, functionally unrelated yet physically contiguous genes on chromosome 11p11.2. Two genes within the ~ 2.1 Mb PSS genomic interval have been shown to contribute to two major PSS phenotypes: *EXT2* (MIM 608210) [1] for multiple exostoses and *ALX4* (MIM 605420) [2, 3] for biparietal foramina. We reported the association of a third gene, *PHF21A* (MIM 608325), with ID and craniofacial anomalies, by observing its truncation in three unrelated individuals with balanced translocations and by comparative deletion mapping in other individuals with PSS [4].

We have previously shown that the balanced chromosomal translocations truncating *PHF21A* in two unrelated patients resulted in the relaxation of repression of the KDM1A target neuronal gene, *SCN3A*, by reducing KDM1A occupancy at the *SCN3A* promoter. This observation led us to conclude that the disruption of *PHF21A* resulted in transcriptional misregulation. We then showed that the suppression of *phf21a* in zebrafish resulted in neuronal apoptosis and craniofacial anomalies, providing additional evidence that PHF21A deficiency was responsible for the ID and craniofacial anomalies observed in our patients with balanced translocations [4]. This implication of *PHF21A* in ID and craniofacial anomalies was further supported by the discovery of two more multigenic microdeletions encompassing this gene in two independent patients with a similar phenotype [5, 6] and one microdeletion at 11p11.2 that does not include *PHF21A* in a patient without ID [7].

During the preparation of this manuscript, three individuals carrying de novo truncated variants within *PHF21A* have been published. Among three patients with ID and craniofacial anomalies, epilepsy was present in case 1, autism spectrum disorder (ASD) in case 2, and overgrowth with macrocephaly in cases 1 and 3. The authors have concluded that PHF21A haploinsufficiency results in ID and craniofacial anomalies, and possibly contributes to ASD, epilepsy, and overgrowth [8]. Here, we report seven individuals with heterozygous pathogenic sequence variants within the coding region of *PHF21A* and provide detailed clinical descriptions, further expanding the phenotype associated with PHF21A haploinsufficiency.

## Methods

### Cell culture

Blood samples derived from patients and their parents were used to establish lymphoblastoid cell lines as described by Nishimoto et al. [9].

### Genomic DNA extraction

Isolation of genomic DNA from human blood was carried out using a standard phenol-chloroform protocol with minor modifications or the Qiagen automated extraction procedure, following the manufacturer’s recommendations.

### Whole exome sequencing

Whole exome sequencing in trios, followed by Sanger sequencing of *PHF21A* variants, was performed on a clinical basis for four of the seven patients. For the paired-end pre-capture library procedure, genomic DNA was fragmented by sonicating and ligating to the Illumina multiplex PE adapters. The adapter-ligated DNA was then PCR amplified using primers with sequencing barcodes. For the target enrichment exome capture procedure, the pre-capture library was enriched by hybridizing to biotin-labeled VCRome 2.1 in-solution exome probes [10] at 47 °C for 64–72 h. For massively parallel sequencing, the post-capture library DNA was subjected to sequence analysis on Illumina HiSeq platform for 100 bp paired-end reads [11]. The following quality control metrics of the sequencing data were generally achieved: > 70% of reads aligned to target, > 95% of the target bases covered at > 20×, 85% of the target bases covered at > 40×, mean coverage of target bases > 100×, and SNP concordance to genotype array > 99%.

### Targeted gene panel sequencing

Five hundred and twenty genes associated with ID/cognitive impairment (Additional file 1: Table S1) were analyzed in trios (propositus, mother, and father) from a cohort of 947 patients referred for ID. These genes were selected by their involvement in ID from a literature search on PubMed. Sequencing with this targeted gene panel was performed for patients 2, 4, and 6.

SeqCap EZ Capture kit (Roche) and 75 bp paired-end reads sequencing on a Nextseq550 Illumina sequencer were performed following the manufacturers’ recommendations. Greater than 95% of target bases were routinely covered at > 30×. Parentage was confirmed using several rare inherited variants.

For two GeneDx cases of patients 3 and 7, the SureSelect Human All Exon V4 (50 Mb) and the IDT xGen Exome Research Panel v1.0 kits were used".

### Sanger sequencing

All variants identified by whole exome sequencing were confirmed by Sanger sequencing of the genomic DNA extracted from the blood of patients. Primer pairs encompassing the individual sequence variants were designed for Sanger sequencing. PCR reactions were carried out in a total volume of 50 μl using GoTag® Green Mastermix (Promega). The conditions for PCR included an initial denaturation at 95 °C for 4 min, 40 cycles consisting of a denaturation at 95 °C for 30 s, annealing at 60 °C for 1 min, and extension at 72 °C for 1 min followed by a final extension for 5 min at 95 °C. Approximately 5 μl of the reaction product was run on a 1% agarose gel containing ethidium bromide (100 mg/ml). The PCR products were purified by a standard protocol and sequenced using the Big Dye® Version 3.1 Cycle Sequencing kit (Applied Biosystems) after confirming the sizes of the amplicons.

### Real-time PCR

Isolation of total RNA from lymphoblastoid cell lines was performed using the RNeasy Plus Mini kit (Qiagen) following the manufacturer’s protocol. Total RNA from the human brain and fetal brain, as well as other tissues (Human Total RNA Master Panel II, Cat# 636643, Clontech), were also used for RT-qPCR. The cDNA synthesis was performed from 1 μg of total RNA using the RevertAid First cDNA Synthesis Kit (Thermo Scientific) following the manufacturer’s instructions. Real-time PCR was carried out in a 20-μl reaction volume containing 2 μl cDNA, 2.5 μM primer, and 10 μl FastStart DNA Green Master (Roche).

### RT-PCR

RNA was transcribed into cDNA with the QuantiTect Reverse Transcription Kit (Qiagen). Resulting cDNA was amplified using PCR primers (available upon request) by PrimeStar GXL (Takara) following the manufacturer’s instructions.

### MiSeq sequencing

RT-PCR products were pooled. Nextera XT DNA Preparation kit (Illumina) was used to create a DNA library, which was fragmented and tagged with adaptors according to the manufacturer’s protocol. Samples obtained were pooled and sequenced in MiSeq (Illumina). Resulting data were aligned to the human genome reference sequence (GRCh37/hg19) using BWA (Burrows-Wheeler Aligner; v0.7.6). The Genome Analysis Toolkit (GATK; v2.1-10) enabled indel realignment and base quality score recalibration. Variants with a quality score > 30 and alignment quality score > 20 were annotated with SeattleSeq SNP Annotation.

### Protein-DNA modeling

We modeled the AT Hook region using the one-to-one threading expert mode platform of Phyre2 [12]. The AT Hook-DNA complex structure was used as a template (PDBID: 2EZE). The chain A within 2EZE having the peptide (25 amino acids long) of the AT Hook region was aligned and used for final modeling. Protein-DNA modeled structures were analyzed, and figures were generated using PyMOL (W.L. DeLano, The PyMOL Molecular Graphics System, 2014, version 1.8, Schrödinger LLC: http://www.pymol.org.10.1038/hr.2014.17.)

## Clinical reports

### Patient 1 (c.1955delC, p.Pro652LeufsX104)

Patient 1 is a 13-year-and-6-month-old Caucasian female with a history of ID, ASD, focal epilepsy, motor apraxia, attention deficit hyperactivity disorder (ADHD), and dysmorphic features. She was born full term by spontaneous vaginal delivery and weighed 4 kg (99th centile). Around 4 months of age, she was noted to have hypotonia, specifically poor head and neck control. She sat independently at around 8 months, walked at 21–22 months, and spoke her first words at 18 months, but was not speaking full sentences until 5 years of age. She currently speaks both English and Romanian fluently. She repeatedly displayed absent reflexes on examination prompting an electromyogram, nerve conduction studies, and an MRI of the brain and spine. The MRI of the spine revealed a low-lying conus but no tethered cord. The remaining studies were normal.

Neurobehavioral concerns began at 10 months of age when she displayed repetitive movements and wringing of her hands. At 3 years and 11 months, she was diagnosed with pervasive developmental disorder-not otherwise specified (PDD-NOS) (Table 1). Around 5 years of age, she exhibited muscle twitching. She had two events at 9 years of age in which she was described to have had altered awareness or was unresponsive. During the same year, she fell limp and had eye deviation, weakness, altered mental status, smacking of lips, and vomiting. A physical exam revealed facial droop, unbalanced waddling gait, and perseveration. An electroencephalogram (EEG) showed intermittent focal slowing in the right temporal area. She was diagnosed with complex partial seizures, which have been well controlled with oxcarbazepine. She was also diagnosed with ADHD. Her most recent neuropsychological testing revealed that her Wechsler Intelligence Scale for Children-Fifth Edition (WISC-V) full-scale score was very low (first centile). She scored poorly across visual spatial fluid reasoning, working memory, and processing speed indexes but performed in the high average range on the verbal comprehension index (77th centile). She continues to struggle with independent planning and organization and becomes frustrated with challenging tasks. She has few close friends and exhibits immature social skills. She has a significant anxiety disorder and continues to progress, but her deficits in attention and executive function continue.

**Table 1:** Clinical features of patients with the mutations of *PHF21A*

	Patient 1	Patient 2	Patient 3	Patient 4	Patient 5	Patient 6	Patient 7
Age	13 years and 6 months	3 years and 4 months	9 years and 9 months	10 years	18 years	6 years	18 years
Sex	Female	Male	Female	Male	Male	Male	Female
Exon	18	13	18	17	15	17	18
Nucleotide change NM_001101802.1	c.1955delC	c.1285G>A	c.1956delT	c.1738C>T	c.1471dupT	c.1738C>T	c.2024delA
Effect on protein NP_001095272.1	p.Pro652LeufsX104	p. Gly429Ser	p.Pro652ProfsX104	p.Arg580Ter	p.Cys491LeufsX81	p.Arg580Ter	p.Gln675ArgfsX81
Inheritance	De novo	De novo	De novo	De novo	Not found in mother	De novo	De novo
Developmental delay	+	+	+	+	+	+	+
Intellectual disability	+	+	+	+	+	+	+
Facial dysmorphism	+	+	+	+	−	+	+
Cranial anomalies	Macrocephaly	−	−	Plagiocephaly	−	−	Macrocephaly
Autism	+	N/A	−	−	+	−	+
Epilepsy/seizures/spasms	+	+	+	+	−	−	−
Language delay	+	+	+	+	+	+	+
Tapering fingers	+	+	+	−	−	−	−
Clinodactyly	+	+	+	−	+	−	−
Syndactyly	−	+	−	+	−	−	−
Impaired motor skills	+	+	+	+	+	+	+
Hypotonia	+	+	+	N/A	−	−	−
ADHD	+	N/A	+	+	+	−	N/A
Anxiety disorder	+	N/A	+	N/A	−	+	+
Neurobehavioral problems	+	+	+	+	+	−	+
Obesity	+	−	+	−	+	−	+

*N/A*, not available. Minus sign (“−”) represents the absence of the corresponding phenotype

**Fig. 1:**
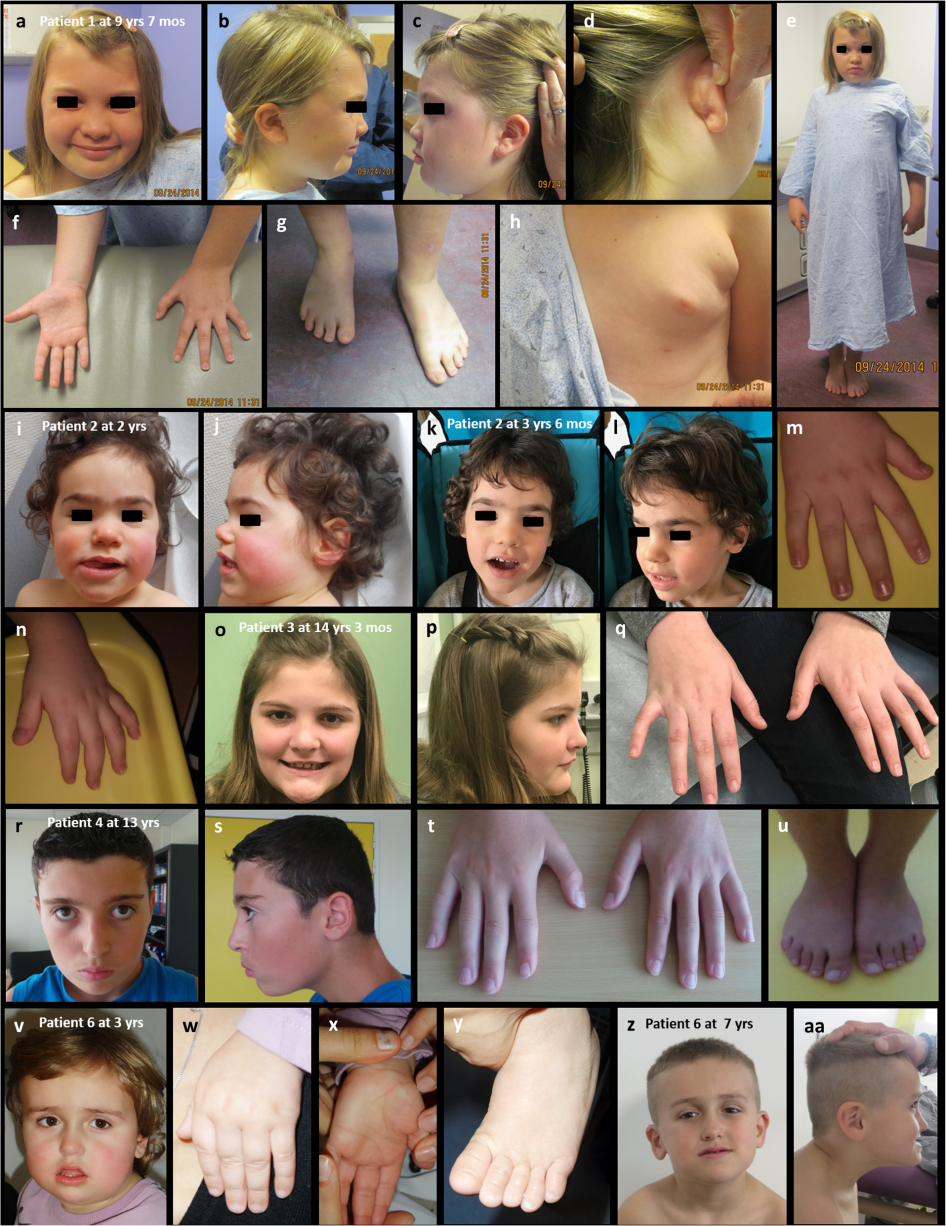
Facial, limb, and full body pictures of patients with *PHF21A* mutations. **a** Facial picture of patient 1 showing a broad nasal bridge, broad nasal tip, and significant bilateral epicanthal folds. **b** Profile view showing a café-au-lait spot on the right cheek, and her columella was wide, short, and hypoplastic. **c** Profile showing a Darwinian tubercle on the low-set left ear. **d** Fleshy and posteriorly rotated ears. **e** Full body image showing truncal obesity. **f** Hands showing mild tapering fingers and clinodactyly. **g** Feet were completely flat and showed brachydactyly of the toes. **h** Inverted nipple on the left and left-sided polythelia. **I**–**L** Patient 2 with a round face, a high forehead, a broad nasal bridge, mild bitemporal narrowing, synophrys, and macrostomia with conical teeth. **m**, **n** Tapering fingers and clinodactyly in patient 2. **O**, **P** Face showing a thinner upper lip and a prominent chin in patient 3. **q** Patient 3 showing tapering fingers and clinodactyly. **r**, **s** Patient 4 with mild plagiocephaly and hypertelorism. **t** normal fingers of patient 4. **u** Minor syndactyly of toes two and three in Patient 4. **v**–**aa** Patient 6 showing midface hypoplasia, a thin upper lip, and a prominent chin in **v** and **z**, **aa**

At 11 years of age, she weighed 54.9 kg (95th centile) with a height of 159.6 cm (> 97th centile) and head circumference of 58 cm (> 97th centile). Her mother has a head circumference of 59 cm (> 97th percentile), and by observation, her father had a large-sized head. She had abundant scalp hair and a very pronounced widow’s peak with a receding anterior hairline laterally. Dysmorphic features included significant bilateral epicanthal folds, a broad nasal bridge, a broad nasal tip (Fig. 1a), one café-au-lait spot (Fig. 1b), several moles on her head below the hairline, and low-set and posteriorly rotated ears, which appeared very fleshy (Fig. 1b–d). She had a Darwinian tubercle (Fig. 1) on the left ear and an ear length of 6 cm (50th centile). Her columella was wide, short, and hypoplastic (Fig. 1a–c). She has mild tapering fingers and clinodactyly (Fig. 1f). Her hand measurements were 16 cm and 15 cm (60th centile), while her feet were at the 75th centile and appeared completely flat. She displayed brachydactyly of the toes (Fig. 1g). She had a hoarse voice, kept her mouth open, and sucked her thumb. Her nipple was inverted on the left, and she had a supernumerary nipple on the same side (Fig. 1h). She was obese with a BMI of 22 (94th centile) (Fig. 1e).

Other tests completed for this patient include an extensive metabolic workup, chromosome analysis, 15q subtelomeres, oligoarray/SNP analysis, methylation studies for Prader-Willi and Angelman syndromes, and an evaluation of the following genes: *MELAS, MERRF, NARP, MECP2, PTEN, TSC1*, and *TSC2*.

### Patient 2 (c.1285G>A, p. Gly429Ser)

Patient 2 is a 3-year-and-4-month-old Caucasian male born to non-consanguineous parents. He has an older brother without any medical issues and no significant family history. He was born at full term after an uneventful pregnancy. At birth, his weight was 3.86 kg (84th centile), his length was 54 cm (99th centile), and his head circumference was 35 cm (66th centile). His initial development was normal. At 11 months of age, he had his first seizure episode with flexor spasms. An electroencephalogram showed hypsarrhythmia. Both the clinical presentation and EEG improved under treatment with vigabatrin. At 20 months, he was placed on combination therapy (with vigabatrin and valproate) due to worsening epilepsy. He was referred to genetics at 24 months of age. At that time, it was noted that the number of spasms had decreased from one episode per day at 11 months of age to two episodes per month, with each episode being a few seconds in duration. The treatment at that time was vigabatrin 90 mg/kg/day.

At 24 months, he weighed 15.5 kg (> 97th centile), with a height of 95 cm (99th centile) and a head circumference of 50 cm (90th centile). Psychomotor milestones were delayed, sitting at 10 months, walking at 34 months, and language limited to monosyllables and reduplication of the same syllable. He displayed symptoms of behavior disturbance, including motor agitation and frequent screams. At 40 months, there was no sign of ASD reported. There were no sleeping or eating difficulties, no history of regression, and no stereotypic movement. He had a round face with a high forehead, a broad nasal bridge, mild bitemporal narrowing, synophrys (without other excess of body hair), macrostomia with conical teeth, normal ears, and a normal palate (Fig. 1i–l). He had clinodactyly on both hands (Fig. 1m–n) and syndactyly of the second and third toes on both feet. He also had myopia. Radiographic evaluation—including an abdominal ultrasound; x-rays of the rachis, pelvis, and limbs; and a brain MRI—was normal. A first-tier metabolic screening was normal (amino acid chromatography in blood and urinary oligosaccharides and mucopolysaccharides). As no specific disease could be suspected on the basis of the clinical presentation, no targeted genetic screening was performed, and whole exome sequencing was proposed to the parents. This patient was identified through GeneMatcher [13].

### Patient 3 (c.1956delT, p.Pro652ProfsX104)

Patient 3 was initially evaluated in a neurogenetics clinic at 9 years and 9 months of age for seizures, ID, and a behavior disorder. She was born full term after an uncomplicated pregnancy and delivery to a G4P4 mother. Her birth weight was 3.4 kg (64th centile), and no perinatal complications were noted. She was developmentally delayed, sitting at 7 or 8 months; combat crawling at 12 months, which evolved to a four-pronged crawl; and walking at 19 months. With regard to language development, she babbled between 8 and 10 months and developed single words by 12 to 18 months of age.

Behavioral concerns began fairly early. At the age of two, she was biting other children and having crying fits. She was started on fluoxetine at about 4 years of age with some improvement in behavior. At the age of five, the behavior concerns persisted, and she began to have episodes related to seizures, consisting of repeated eye blinking and eye rolling that lasted for up to hours at a time. During this time, she also had poor sleep. She was therefore referred to a neurologist. An EEG, done through an epilepsy monitoring unit, revealed seizure activity precipitated by eye closure that occurred diffusely over the occipital region. She was started on levetiracetam at that time and was concurrently treated with fluoxetine, guanfacine, and lisdexamfetamine for behavioral issues. At the age of 9 years and 9 months, she was having the eye blinking episodes for longer than 10 min approximately two to three times a week for which lorazepam was given.

Her past medical history is relatively non-contributory for major illness or complications. Due to concern for loss of cognitive skills at the age of 9 years, a brain MRI was performed which was normal. A neurological examination of the patient was normal without focal deficits, both centrally and peripherally. She exhibited a mostly slow and wobbly gait, being unsteady at times. Her speech was robotic with quick short responses. She had a thinner upper lip, a prominent chin (Fig. 1o, p), clinodactyly, and tapering fingers on both hands (Fig. 1q). At the age of 12 years and 5 months, it became apparent that the peak in behavioral episodes and the seizure activity occurred at a time just prior to the onset of her menstrual cycle. She continued to have interrupted sleep during the night. Normal testing included chromosomal microarray, fragile X, plasma amino acid analysis, urine organic acid levels, and pyruvate levels. Family history was non-contributory. Due to the continued clinical concerns, whole exome sequencing was pursued for the patient.

### Patient 4 (c.1738C>T, p.Arg580Ter)

Patient 4 is a 10-year-old male with no family history of neurodevelopmental disorders. The patient experienced psychomotor retardation with sitting at 12 months and walking at 27 months. He was also noted to have language and fine motor delays. At the age of 6 years, he was diagnosed with mild ID including difficulties with reasoning and abstraction, attention deficit disorder, and verbal and visual-constructive dyspraxia. He also had hip dysplasia, valgus feet, mild plagiocephaly, hypertelorism (Fig. 1r–s), and minor 2–3 toe syndactyly (Fig. 1u). The patient had multiple generalized tonic-clonic seizures at 9 years of age. Interictal EEG was normal, but a seizure was recorded in the left temporal region, for which he was treated with valproate. At the age of 10, the patient experienced cognitive regression following an episode of partial seizures, gaze fixation, and gestural automatism. An EEG recording showed diphasic spikes in the two posterior regions, which diffused into the anterior regions and produced continuous waves of 20 to 40 s during sleep. Levetiracetam therapy did not control the seizures, and thus, clobazam was prescribed with partial alleviation.

A brain MRI at age nine was normal. Fragile X testing, a chromosome analysis performed on lymphocytes, and chromosomal microarray were normal. Targeted sequential studies of the *ARX*, *FOXP2*, and *GRIN2A* genes did not find any abnormalities. A panel of 450 ID genes was sequenced, with negative results. Whole exome sequencing in the patient and both of his parents was performed.

### Patient 5 (c.1471dupT, p.Cys491LeufsX81)

Patient 5 is an 18-year-old Hispanic male. He was born at 39 weeks gestation to a 15-year-old G1P1 mother via Cesarean section. The father was 16 years old at the time of birth. Consanguinity was denied. The pregnancy was complicated by possible early exposure to alcohol and marijuana. Birth weight was 3856 g (84th centile), and birth length was 48.3 cm (20th centile). After birth, he was noted to have difficulty breathing, sucking, and swallowing. He required supplemental oxygen and spent 1 week in the neonatal intensive care unit due to hypoxia, feeding difficulties, and jaundice. Over time, he was diagnosed with developmental delay, ID, attention deficit disorder, bipolar disorder, and ASD. He sat without assistance at 5 to 6 months of age, crawled at 7 to 8 months of age, and walked at 18 months of age. He spoke his first words at 1 year of age and began to combine words at 3 years of age. A speech assessment at 4 years of age revealed delayed receptive and expressive language skills—characteristic of a 2 to 3 year old—and poor speech intelligibility. Throughout his education, he was enrolled in special education classes. He had a history of recurrent ear infections requiring placement of multiple sets of pressure equalization tubes. An audiometry evaluation performed at 5 years of age revealed slight conductive hearing loss on the right and normal hearing on the left. An MRI of the brain performed at 5 years of age was normal. He was later diagnosed with obesity and obstructive sleep apnea.

At 17 years and 10 months of age, his height was 171 cm (26th centile), his weight was 115 kg (99.5th centile), and his body mass index was 39.3 (99.6th centile). He was non-dysmorphic, but was noted to have large earlobes and bilateral fifth finger clinodactyly (Table 1). A previous workup included chromosome analysis, a microarray-based copy number variant analysis, plasma amino acids, urine organic acids, ammonia, *NSD1* sequencing, and fragile X testing, all of which were normal. Whole exome sequencing was performed on a clinical basis.

### Patient 6 (c.1738C>T, p.Arg580Ter)

This child is the third boy of four, born to non-consanguineous parents of French and Tunisian ancestry. He was born at 38 weeks gestation by Cesarean section because of macrosomia. His birth parameters were 4610 g for weight, 52 cm for length, and 38 cm for head circumference. At birth, he had no hypotonia or feeding difficulties. He sat at 17 months, walked independently at 26 months, and spoke his first words at 2 years and first phrases at 5 years. Neuropsychological evaluation at 4 years and 5 months (WPPSI IV) showed global developmental delay, with skill levels equivalent to a 2-year and 6-month to a 3-year and 4-month level. He needed aid at school and entered a special school for intellectually disabled children at 5 years and 9 months. He had recurrent episodes of otitis media and a tendency to be constipated, but no seizures, regression, sleeping difficulties, or behavior disorders were reported. A brain MRI was normal, as well as a renal ultrasound. Standard biochemical and metabolic tests were normal as were fragile X testing and SNP array.

At last examination, at 5 years and 3 months, the patient had a weight of 23.7 kg (+ 2.5 SD), a height of 113 cm (+ 1 SD), and a head circumference of 54.2 cm (+ 2 SD). He had midface hypoplasia, thin upper lip, and prominent chin (Fig. 1v, z). He had no noticeable distinctive features of his hands and feet, and no skin lesions. Both testes were palpable. His neurologic examinations were normal. A targeted panel of 520 genes associated with ID/cognitive impairment was performed.

### Patient 7 (c.2024delA, p.Gln675ArgfsX81)

At the time of assessment, patient 7 was an 18-year-old Asian female, the first-born child of non-consanguineous Hmong parents, who subsequently had four additional healthy, developmentally normal children. She was born at full term after an uneventful pregnancy. At birth, her weight was 3.062 kg (35th centile), her length was 48.3 cm (32nd centile), and her head circumference was not available. Her early motor development was normal. She had delayed speech with first words at 18 months, followed by additional developmental delays and eventual development of cognitive impairment. At 3 years, she was diagnosed with PDD-NOS, due to loss of social and behavioral skills starting around 2 years of age, sensitivity to loud sounds, impaired coordination, and difficulties with balance.

She had a history of behavioral aggression throughout her schooling, particularly with regard to ownership and sharing, which intensified between 16 and 17 years of age, associated with diagnoses of obsessive-compulsive disorder and anxiety. She had one acute out-of-character behavioral episode, characterized by disinhibited behavior and unusual delusions, without hallucinations, lasting a few days at age 17, for which brain MRI, 24-h continuous EEG, and subsequent neurological evaluation were negative. She has never had any observed seizures.

In an assessment at 18 years, she was noted to need substantial help with self-care activities and could not understand or follow instructions. Her interactions and behaviors were immature, characterized as a 4-year-old level by her school evaluations. She had short stature and obesity (weight 88.2 kg, > 98th centile; height 153.0 cm, 6th centile; BMI 37.7 kg/m^2^, 98th centile) with macrocephaly (head circumference 58 cm; > 98th centile) (Table 1). She had a history of continuously gorging or binge eating to the point of vomiting, requiring her parents to restrict her food intake.

She was mildly dysmorphic, with sparse lateral eyebrows, telecanthus, left preauricular pit, prominent cupid bow configuration of the upper lip, and slack facial expression. She had numerous self-inflicted, skin-picking lesions on her arms, in various stages of healing, and one hyperplastic keloidal scar elsewhere. Her speech was fluent but very simple, with sound substitution errors very typical of a young child. There was periodic echolalia, in addition to outbursts of out-of-context speech, without regard to interrupting other speakers. Negative genetic testing included Prader-Willi/Angelman syndrome methylation and MLPA, fragile X, and CNV analysis via SNP microarray. However, areas of homozygosity were identified across multiple chromosomes, with an overall percentage of autosomal/genomic homozygosity of 2.5%. No candidate recessive genes were identified within the homozygous regions. As no specific disease could be suspected on the basis of the clinical presentation, family trio sequencing of GeneDx’s Autism/ID Xpanded Panel with 2308 genes was performed, using oral rinse samples from her and both parents. This patient was identified through GeneMatcher [13].

## Results

**Fig. 2:**
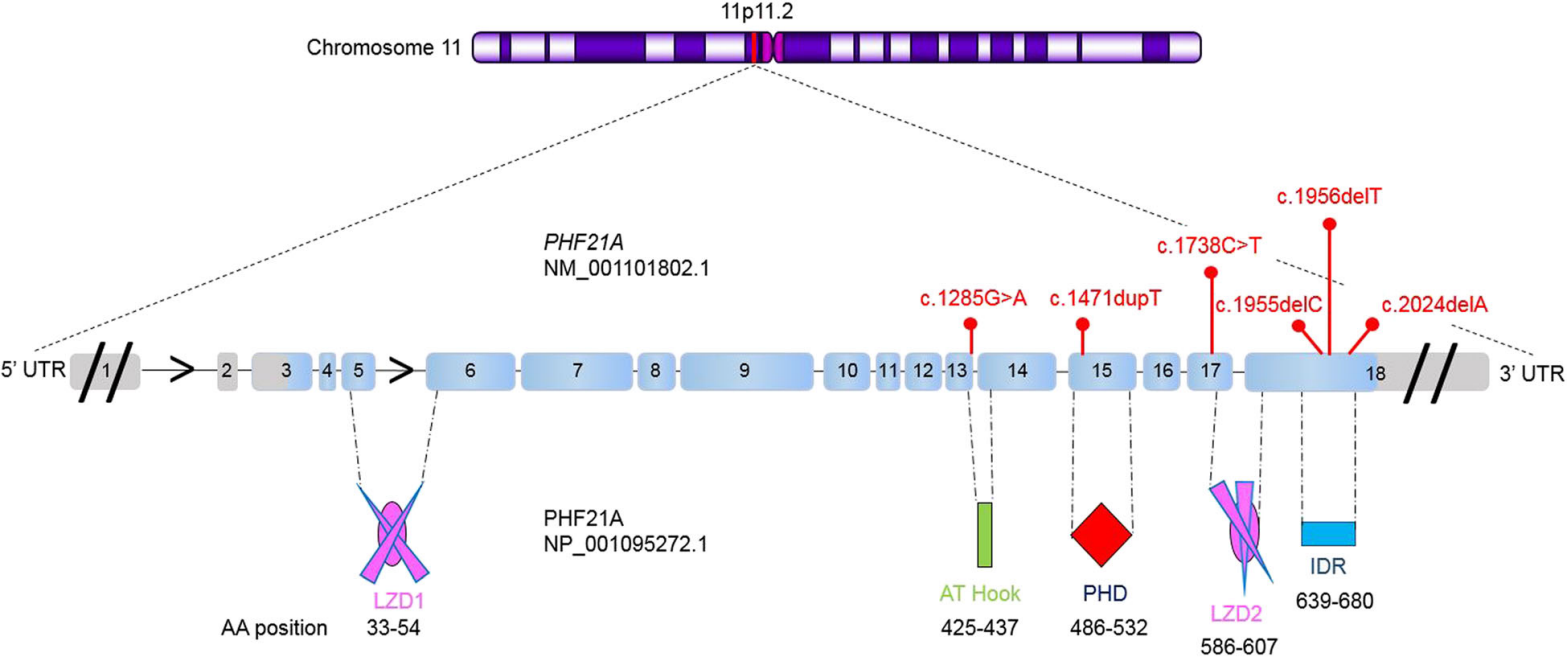
Mutations in *PHF21A* and domain structure of the protein. Six mutations located in corresponding exons are depicted. Eighteen exons are represented by light blue boxes with corresponding numbers below connected by a horizontal black line representing the introns. The arrow in the intron shows the transcription direction. The UTRs are depicted by gray boxes, and the diagonal lines indicate not to scale. Note that the size of exons, mutation location, and protein domains are to scale; however, the size of the introns and UTRs are not. The identified deletions, duplication, and point mutations on the cDNA level (NM_001101802.1) are depicted in red above the exons. The nonsense mutation c.1738C>T has been found in two patients. The dotted black lines connect the corresponding exons encompassed within each functional domain demarcated by a corresponding amino acid residue number (NP_001095272.1)

**Fig. 3:**
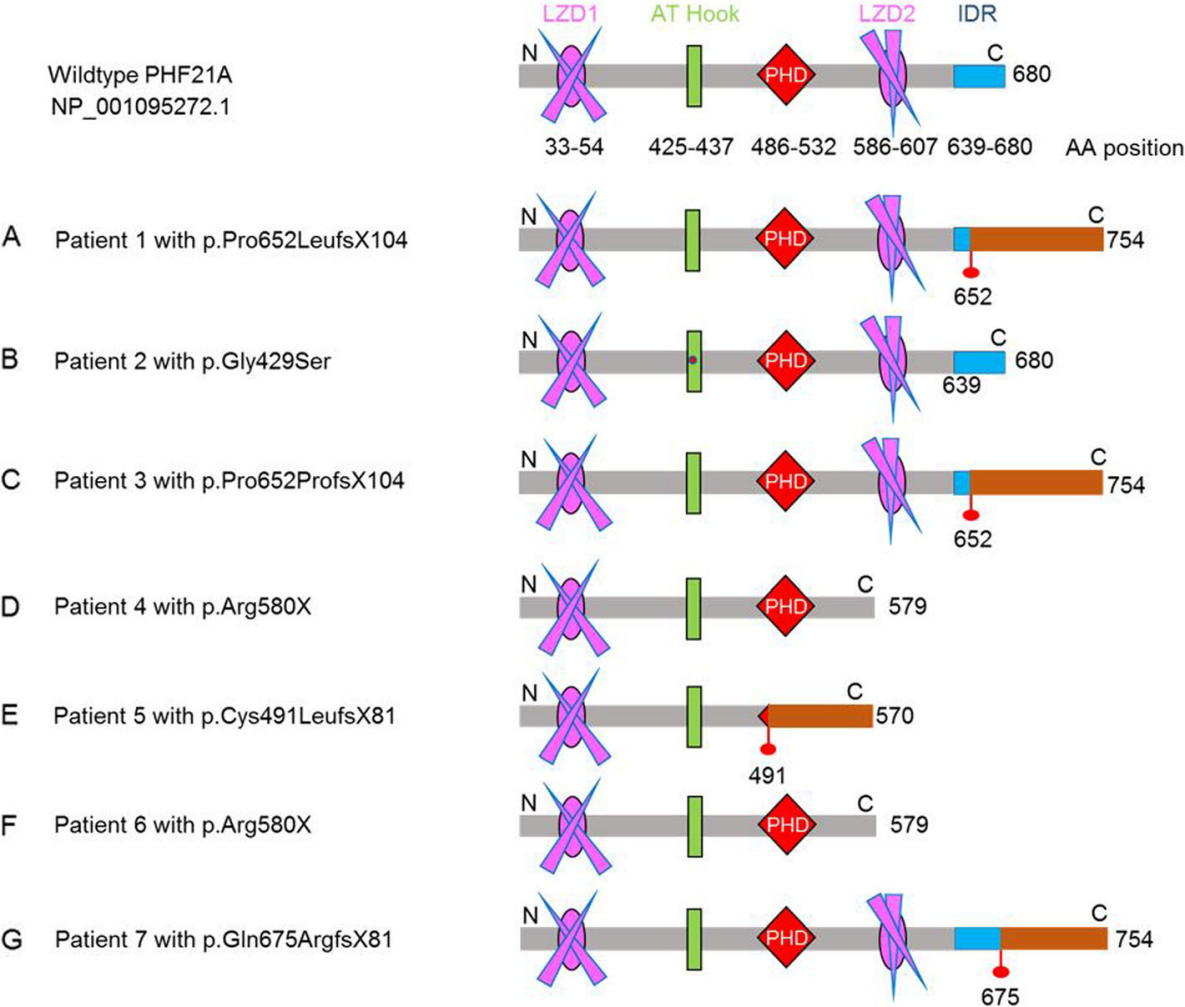
PHF21A functional domains in wildtype and theoretical missense and truncated or altered proteins in seven subjects. PHF21A contains two leucine zipper domains (LZD1 and LZD2), one AT Hook domain, one PHD zinc finger domain, and one intrinsically disordered region (IDR). The amino acid positions of all domains are indicated as numbers below the domain structures. Aberrant amino acid sequences produced by frameshifts are indicated as brown bars, under which the starting aberrant residue is indicated as a number. Note that the size of the functional domains is to scale; however, the regions connecting them are not. In patient 1, the preserved 651 amino acids are followed by 103 aberrant amino acids, which results in an aberrant elongated protein of 754 aa. In patient 2, the missense mutation indicated as a red dot is located in the AT Hook domain. In patient 3, the first 652 amino acids in the wildtype protein were followed by 102 aberrant amino acids, resulting in a 754 aa elongated mutant protein. A 754 aa elongated chimeric protein in patient 7 is composed of the 674 aa wildtype protein plus 80 aberrant amino acids. If expressed, the truncated protein in patients 4 and 6 loses a LZD2 domain, and in patient 5, the LZD2 domain as well as the PHD finger domain, both essential for binding H3K4me0, is missing. In patients 1, 3, and 7, the IDR has been truncated as shown by the partial blue box

Using whole exome sequencing or ID gene panel sequencing, we identified six unique variants in seven patients. Four were frameshift variants, three of which are de novo and potentially produced aberrant elongated proteins (in patients 1, 3, and 7), while the fourth (in patient 5) could produce a shortened protein that also has the addition of 80 aberrant amino acids. Two patients (4 and 6) have identical de novo nonsense variants, and the last was a de novo missense variant (patient 2) (Table 1, Figs. 2 and 3). Sanger sequencing confirmed the presence of all variants in the probands and the absence of the variants from the available parents.

In patients 1 and 3, we identified a de novo c.1955delC variant (NM_001101802.1) causing a frameshift (p.Pro652LeufsX104) and a de novo c.1956delT variant causing a frameshift (p.Pro652ProfsX104), respectively, both in exon 18 and predicted to produce 754-amino-acid (aa) elongated proteins compared to the wildtype PHF21A of 680 aa (NP_001095272.1) (Fig. 3 (A, C)). As a result of the frameshift in patient 1, this chimeric protein is 74 aa longer, comprised of the N-terminal 651 aa of wildtype PHF21A followed by an aberrant 103 aa (aa 652–754). In patient 3, the mutant protein has an aberrant 102 aa sequence (aa 653–754), same as patient 1, except for a difference at the 652nd residue.

**Fig. 4:**
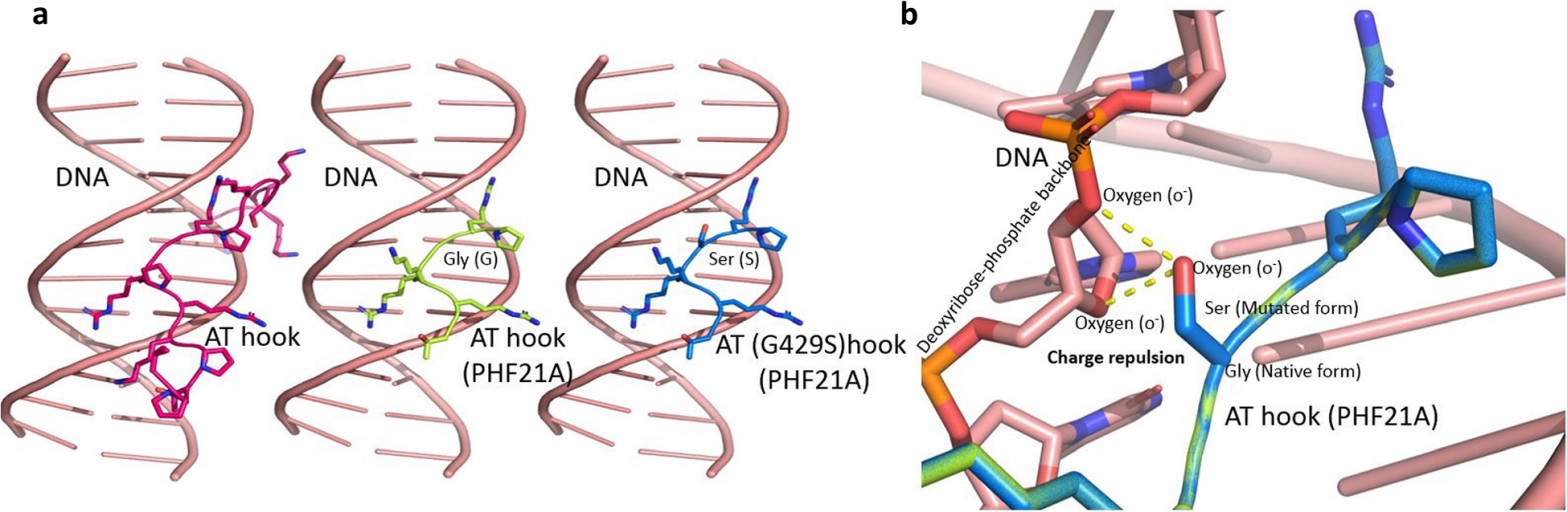
**a** Structure of AT Hook: DNA complex (left panel) represented in ribbon diagram (PDBID: 2EZD). AT Hook motif in pink interacts with the minor groove of DNA in salmon. AT Hook motif in lemon of PHF21A was modeled (middle panel) based on the PDBID: 2EZD. The mutated AT Hook (Gly429Ser) motif in marine was similarly modeled (right panel) based on the PDBID: 2EZD. **b** Superposition of the modeled structure of AT Hook region in native form (Gly) and in mutated form (Ser). The presence of serine in the mutated form creates a charge repulsive environment, which is not conducive to protein-DNA interaction

In patient 2, we found a de novo missense variant c.1285G>A at the last nucleotide of exon 13 leading to an aa substitution p.Gly429Ser (Fig. 2), at a highly conserved residue within the AT Hook domain, which binds AT-rich DNA (Fig. 3 (B)). It is predicted to be deleterious using MutationTaster and SIFT but not by Polyphen2. Because the variant is predicted to alter donor splice scores (SFF score 75.43 versus 87.56; MaxEntScan 3, 71 versus 8, 41 and NNSplice score 0, 43 versus 0, 99), cDNA libraries obtained by RT-PCR from the blood of patient 2 were pooled and sequenced using Illumina MiSeq. It did not reveal any aberrant splice transcripts, indicating this variant did not disrupt the splicing mechanism. In order to understand the structural basis of this mutation (Gly429Ser), we modeled native as well as mutated AT Hook region based on a previously solved structure (PDBID: 2EZD) of AT Hook region complexed with DNA [14] (Fig. 4a). The presence of a hydroxyl group (polar) in the side chain of serine relative to glycine (no side chain) creates a repulsive charge due to its proximity to the negatively charged phosphate backbone of DNA (Fig. 4b).

**Fig. 5:**
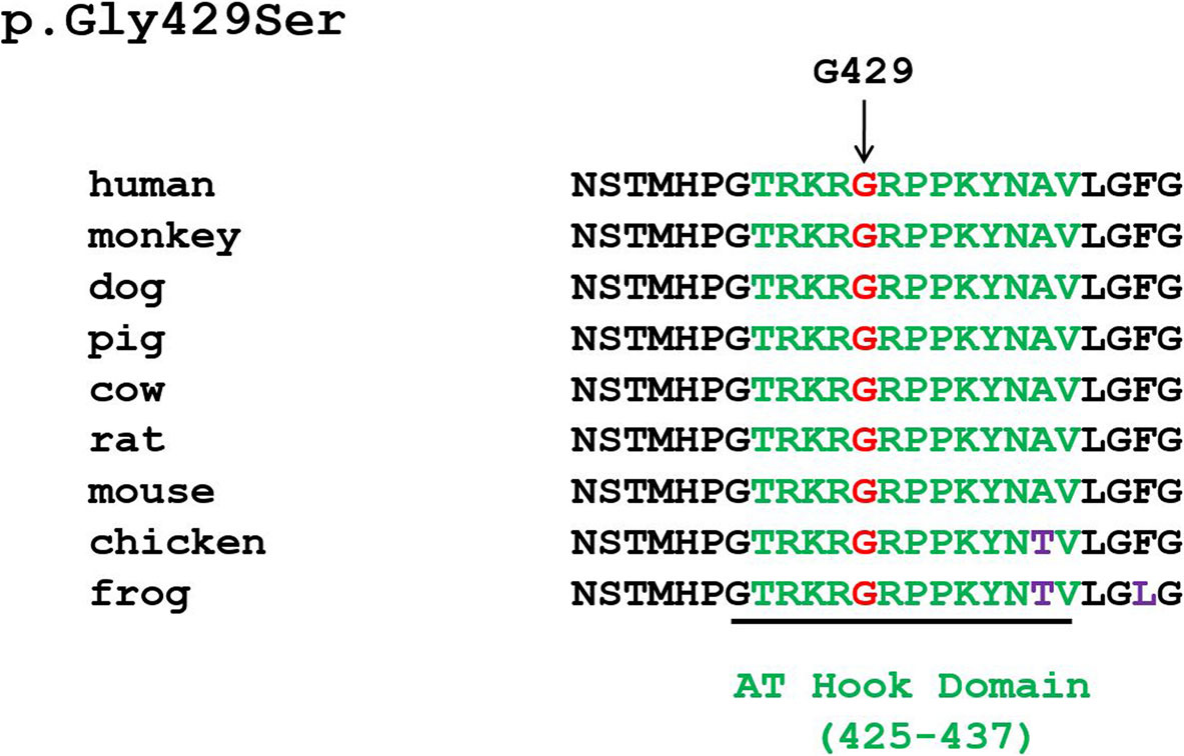
ClustalW multiple alignment of partial protein sequences of PHF21A orthologs. The position of the residue affected by the missense mutation of *PHF21A* in patient 2 is marked by an arrow and a red letter in the corresponding segments of the multiple alignment. The amino acid residues that differ from the sequence of the human PHF21A protein are indicated in violet, and the sequence of the AT Hook domain (aa 425–437 in NP_001095272.1, UniProtKB-Q96BD5) is indicated in green. The mutated amino acid glycine is evolutionarily fully conserved in all nine available PHF21A orthologs

Glycine 429, in the central typical glycine-arginine-proline tripeptide GRP motif [15] of the AT Hook domain, is evolutionarily fully conserved in all nine available PHF21A orthologs (Fig. 5). Given that the other six mutations are nonsense or frameshift mutations leading to loss-of-function, this missense mutation likely results in a non-functional PHF21A mutant. The non-functionality likely arises from the compromise in AT Hook: DNA interactions. The phosphate group (phosphoric acid, PO_4_^2−^) of DNA makes it a weak acid with a negative charge. Much of the AT Hook’s DNA domain/motif is dominated by basic aa residues like lysine and arginine, including its four core Arg-Gly-Arg-Pro (RGRP) residues, which are dominated by a positive charge. Replacement of glycine with serine may likely interfere with the binding of DNA, resulting in a diminished binding affinity, due to the polar mutant serine with a partial negative charge, which would create charge repulsion. This indicates that the AT Hook domain may be critical for the function of PHF21A.

The same de novo nonsense change c.1738C>T in exon 17 was identified in patients 4 and 6 (Table 1, Fig. 2) and creates a premature stop codon (p.Arg580Ter, Fig. 3 (D, F)). The premature stop codon is located 48 nucleotides upstream from the exon 17–18 junction in patients 4 and 6, without satisfying the “> 50–55 nucleotide rule” of more than 50 nucleotides upstream from the last exon-exon junction for nonsense-mediated mRNA decay [16, 17]. Therefore, it is predicted to not elicit nonsense-mediated mRNA decay, and a truncated protein without the LZD2 domain and C-terminal domain would likely be expressed (Fig. 3 (D, F)).

A heterozygous c.1471dupT variant in exon 15 was identified in patient 5 (Fig. 2), which causes a frameshift (p.Cys491LeufsX81), and if translated would create a truncated mutant protein of 570 aa, which is 110 aa shorter than the 680 aa wildtype PHF21A, composed of the N-terminal 490 aa of PHF21A followed by 80 additional aberrant aa (Fig. 3 (E)). The premature termination codon is located 74 bp upstream from the junction of exons 17 and 18, thus likely to trigger nonsense-mediated mRNA decay, preventing the expression of the truncated protein without the PHD finger domain, essential for binding H3K4me0 (Fig. 3 (E)). The c.1471dupT variant was not seen in his healthy mother, but the paternal DNA could not be obtained.

In patient 7, a de novo heterozygous variant (c.2024delA) in exon 18 was identified (Table 1, Fig. 2). It would cause a frameshift p.Gln675ArgfsX81, which would produce an elongated 754 aa mutant protein with the N-terminal 674 aa of PHF21A followed by 80 additional aberrant aa (Fig. 3 (G)).

Although the size and composition of the aberrant PHF21A proteins in patients 1 and 3 are almost identical, varying only by 1 aa at the 652th residue (leucine in patient 1 and proline in patient 3), the respective phenotypes differ. The one nucleotide deletions c.1955delC, c.1956delT, and c.2024delA in patients 1, 3, and 7, respectively, occur far downstream in the last exon (exon 18) (Fig. 2), creating an identical delayed termination codon TAG at the same position in exon 18. This is downstream of the last splice junction (between exons 17 and 18) and therefore does not satisfy the “> 50–55 nucleotide rule” of more than 50 nucleotides upstream from the last exon-exon junction [16, 17]. This finding suggests that the mRNAs in patients 1, 3, and 7 are not likely to be targets for nonsense-mediated mRNA decay. Consequently, the three elongated aberrant proteins would likely be expressed (Fig. 3 (A, C, G)).

We have analyzed the wildtype PHF21A protein sequence for domains in the C-terminal region using SMART (https://smart.embl.de/). Apart from the usual AT Hook region and PHD domain, a low-complexity region at the C-terminal (aa 650–671) tail is apparent. The low-complexity region suggests a disordered but important region for protein function. To obtain a more in-depth understanding, we analyzed the sequence using MobiDB, a database of protein disorder and mobility annotations (http://mobidb.bio.unipd.it/search). The analysis clearly indicated an intrinsically disordered region (IDR) which lies in C-terminal comprising residues 639–680.

### Expression pattern of *PHF21A* in the brain and other tissues

**Fig. 6:**
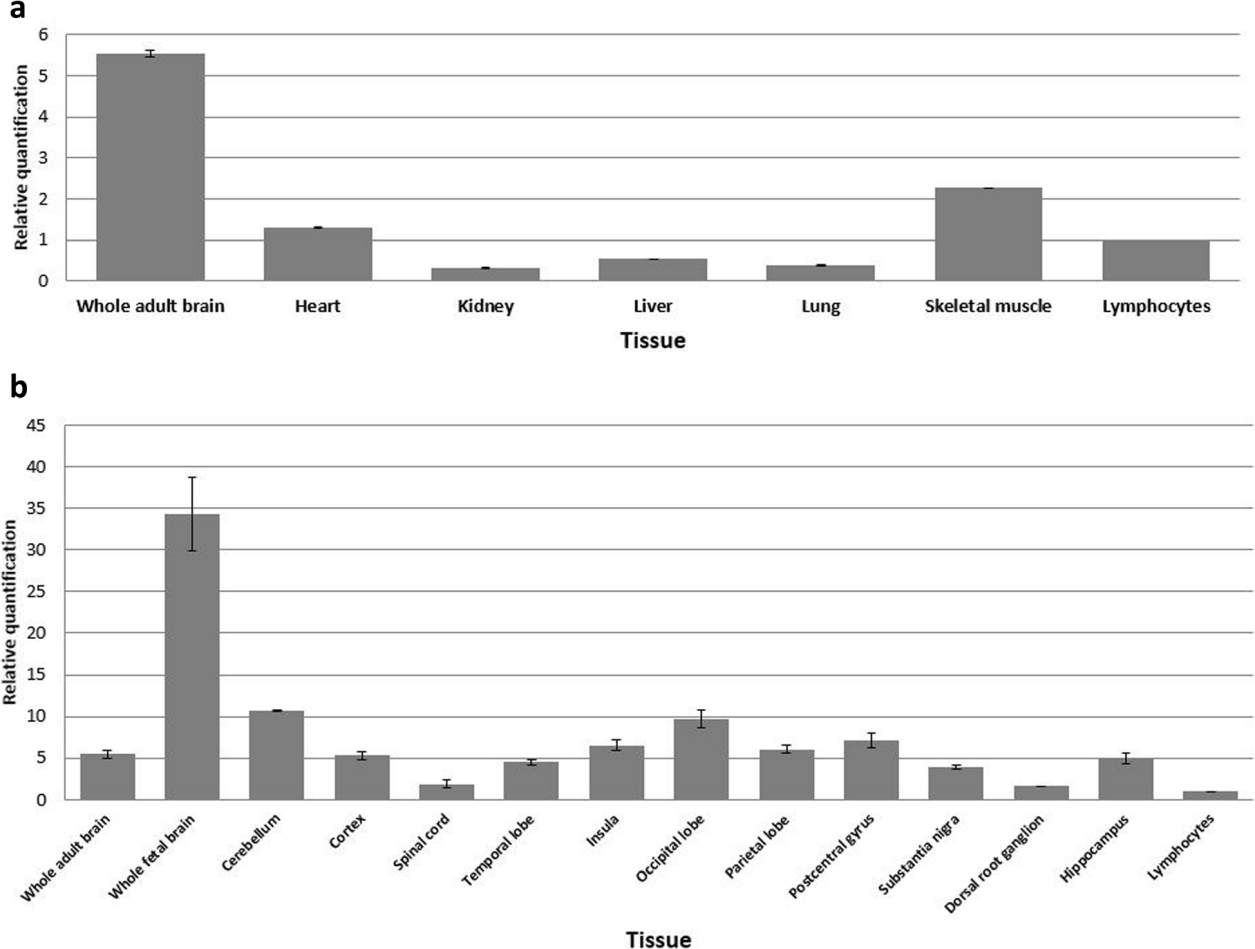
Transcript levels of *PHF21A* in the brain and other human tissue as determined by RT-qPCR. **a** A higher *PHF21A* expression was detected in the adult brain compared to the heart, kidney, liver, lung, skeletal muscle, and lymphocytes. **b** *PHF21A* is abundantly expressed in the fetal brain. High levels of *PHF21A* transcripts were also detected in the cerebellum, occipital lobe, postcentral gyrus, insula, cortex, and hippocampus

We investigated the transcript levels of *PHF21A* by RT-qPCR in whole human brain and other tissues and found that *PHF21A* transcripts were expressed at higher levels in the whole brain compared to other tissues, including the heart, kidney, and lung (Fig. 6a). The transcripts were at least fivefold higher in the brain and twofold higher in the skeletal muscle relative to lymphocytes, supporting the possibility that its mutations can cause the neurological phenotype, impaired motor skills, and hypotonia seen in our patients.

A comprehensive *PHF21A* transcript analysis in different regions of the brain revealed a ~ 34-fold higher expression in the fetal brain relative to lymphocytes. Compared to lymphocytes, levels of *PHF21A* transcripts were tenfold higher in the cerebellum, ninefold higher in the occipital lobe, and fivefold higher in the hippocampus, known to be associated with learning and memory [18]. The spinal cord and dorsal root ganglion expressed lower levels of *PHF21A* transcripts (Fig. 6b). In contrast, very low levels of *PHF21A* expression were detected in the kidney, liver, and lung (Fig. 6a).

## Discussion

Three genes within the 2.1 Mb genomic critical region at 11p11.2 are the major determinants of PSS, characterized by multiple exostoses, biparietal foramina, ID, and craniofacial anomalies. In 1996, *EXT2* was implicated as the cause of multiple exostoses when a 4-bp *EXT2* deletion causing a frameshift was identified in all affected members of a family with multiple exostoses [1]. In 2000, *ALX4* was implicated as the cause of biparietal foramina in PSS with the identification of *ALX4* point mutations: a one-nucleotide deletion and a missense variant in two unrelated families affected with biparietal foramina [3].

Although we reported that *PHF21A* was associated with ID and craniofacial anomalies by the positional cloning of two patients with balanced translocations in 2012 [4], no point mutations affecting this gene had been reported to confirm its deleterious impact until three patients with de novo truncating mutations were reported very recently [8], while this manuscript was being prepared. Taking into account that one out of the three reported patients has ASD [8], we also identified an additional three patients with ASD (patients 1, 5, and 7), further suggesting that *PHF21A* is a novel ASD gene. Patients 1 and 3 have very similar mutations, differing by only one amino acid, yet only patient 1 has ASD. Intriguingly, obesity may be another feature caused by *PHF21A* mutation, seen in three of our patients (patients 1, 3, and 5; Fig. 1e, Table 1), as well as in a female with a balanced translocation and *PHF21A* truncation [4] (Table 1).

The common phenotypes seen in all seven patients reported here are developmental delay, ID, language delay, and impaired motor skills. Seizures were observed in four patients. The existence of novel genes at 11p11.2 for hypotonia [6] and neurobehavioral problems [7] were suggested previously. The hypotonia in three patients and neurobehavioral problems in six patients here suggest that *PHF21A* is a contributory gene for these clinical features in PSS.

The extra C-terminal aberrant tail in the elongated proteins from patients 1, 3, and 7 could act dominant negatively by a gain-of-function mechanism, which might result in a discrepant phenotype from that caused by haploinsufficiency. However, significant phenotype differences were not seen in these patients when compared to the rest of the cohort (Table 1).

We also found a new intrinsically disordered region (IDR) domain at the C-terminus of PHF21A (aa 639–680 in NP_001095271.1). If proteins are produced from the nonsense or frameshift variants in any of our patients, this region would be deleted or truncated (Fig. 3) and thus might have an important function, although additional patients and functional characterization would be needed for further confirmation. IDR domains provide flexibility to the protein and facilitate different conformational requirements for binding modifying enzymes as well as their receptors [19]. These IDRs are particularly prominent within proteins involved in cell signaling, transcription, and chromatin remodeling functions [20, 21]. At a first glance, missense variants in IDR (639–680 aa) at the C-terminus of PHF21A do not seem constrained in gnomAD (Genome Aggregation Database, https://gnomad.broadinstitute.org/). However, among the 32 heterozygous missense variants in the IDR listed in gnomAD, only 4 rare variants (p.Glu678Ala, p.Ser668Arg, p.Ser663Tyr, and p.Thr650Asn), each found in 1 or 2 individuals among over 100,000 people, are predicted as probably or possibly damaging. These findings raise the possibility that among the background of neutral missense variants, some rare missense variants in this region could be pathogenic. Alternatively, the IDR of PHF21A might be intolerant to truncation, but tolerant to missense variants.

The profound expression in the human fetal brain emphasizes the role of PHF21A in early human development, which is consistent with features in our patients, such as developmental delay, ASD, ADHD, and epilepsy observed at an early age (Table 1).

*PHF21A* encodes a protein that specifically binds unmethylated H3K4 as part of a histone demethylase complex that participates in suppression of neuronal gene expression. Histone-modifying enzymes such as histone methyltransferases [22, 23] and demethylases [24–26] have previously been found mutated in syndromic ID. PHF21A is neither a writer nor an eraser, but instead specifically binds unmethylated histone H3K4me0 as a reader in a KDM1A multiprotein complex [27]. This suggests for the first time that a non-catalytic protein targeted to an unmethylated histone site is critical for normal cognitive function and craniofacial development.

Among the other components of the KDM1A demethylase complex, one balanced translocation disrupting *ZMYM3* [28] and one familial missense mutation of *ZMYM3* [29] have been reported. Most importantly, three boys with three different de novo heterozygous missense mutations in *KDM1A* have been reported, sharing craniofacial anomalies, developmental delay, and hypotonia [30]. Furthermore, one patient with KGB syndrome and Kabuki syndrome has been reported to have a de novo missense mutation of *KDM1A*, along with a de novo 3-bp deletion of another gene, *ANKRD11* [31]. Collectively, mutations of four components in the KDM1A demethylase complex have been identified in syndromic ID patients. The parallels between *KDM1A*, *PHF21A*, *ZMYM2* [32], and *ZMYM3* suggest that other genetic loci underlying ID may encode other proteins that participate in protein complexes involving KDM1A or potentially other demethylases.

### Limitations

More functional studies on the AT Hook domain and IDR of PHF21A are required regarding its function in the neurodevelopmental process as a reader of unmethylated histone H3K4me0. Missense or truncating mutations in IDR in human patients need to be found to reinforce its functional role. Since blood from the patients was not available while this manuscript was being prepared, we could not demonstrate that the truncated variants without the IDR are expressed, precluding any definite conclusions about the role of this region.

## Conclusion

In this study, we have identified seven heterozygous coding mutations, among which six are known to be de novo. Four frameshift mutations and one nonsense mutation in two patients are loss-of-function variants. Based on the patient’s phenotype, one heterozygous missense mutation in the AT Hook domain is as deleterious as other mutations and, based on functional predictions, likely to cause loss of PHF21A function. These mutations support the hypothesis that haploinsufficiency is the underlying pathogenic mechanism of PHF21A. The overlapping phenotypes in our patients are developmental delay, ID, language delay, and impaired motor skills. Additionally, some of our patients display ASD, epilepsy, ADHD, anxiety disorder, hypotonia, tapering fingers, clinodactyly, and syndactyly, which extends the clinical features caused by *PHF21A* mutations. Furthermore, we discovered that *PHF21A* is abundantly expressed in the human fetal brain and skeletal muscle, which is consistent with the neurological phenotypes and hypotonia.

## Additional file

Additional file 1: List of 520 genes in the intellectual disability panel. (XLSX 17 kb)

## Data Availability

All data generated or analyzed during this study are included in this article. The primer sequences for Sanger sequencing, Real-time PCR, and RT-PCR will be provided upon request.

## Abbreviations

ADHD: Attention deficit hyperactivity disorder
ASD: Autism spectrum disorder
EEG: Electroencephalogram
ID: Intellectual disability
IDR: Intrinsically disordered region
PPD-NOS: Pervasive developmental disorder-not otherwise specified
PSS: Potocki-Shaffer syndrome
WISC-V: Wechsler Intelligence Scale for Children-Fifth Edition
